# Treatment of Chancre

**Published:** 1895-11

**Authors:** 


TREATMENT OF CHANCRE.—Von Herff, of Halle, treats soft chancre by disinfecting with a strong solution of bichloride of mercury; he then applies pure carbolic acid. If necessary, cocaine may be first applied to lessen pain. Moist compresses of permanganate of potash, sitz baths, and hot permanganate of potash douches are used for four or five days, after which any ulcers that are not already healing may be touched the second time with carbolic acid in the same manner. This method has the advantage that it generally prevents the suppuration of the glandular swellings.—*Modern Medicine.*

